# Template-Free Synthesized Gold Nanobowls Composed with Graphene Oxide for Ultrasensitive SERS Platforms

**DOI:** 10.1021/acs.jpcc.3c03607

**Published:** 2023-08-18

**Authors:** Mateusz Kasztelan, Sylwia Zoladek, Władysław Wieczorek, Barbara Palys

**Affiliations:** † Faculty of Chemistry, University of Warsaw, Pasteura 1, Warsaw 02-093, Poland; ‡ Faculty of Chemistry, Warsaw University of Technology, Noakowskiego 3, Warsaw 00-664, Poland

## Abstract

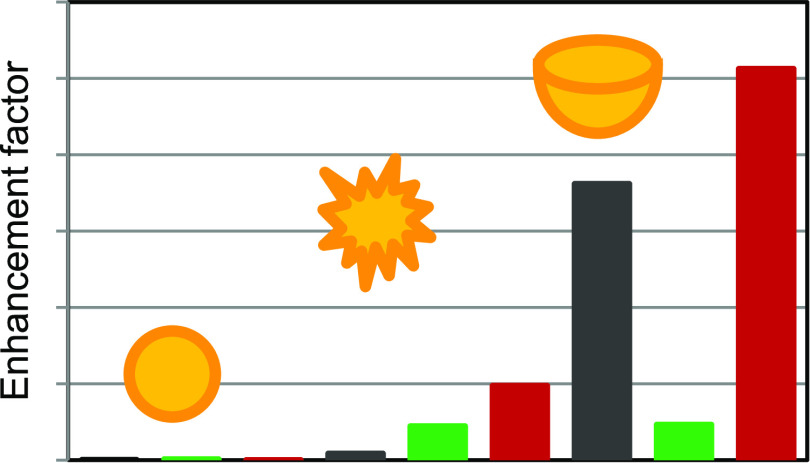

Engineering of plasmonic properties of gold nanostructures expands the field of their applications from photocatalysis and photothermal effects to ultrasensitive surface-enhanced Raman spectroscopy (SERS). The known methods of preparation of gold nanobowls involve the deposition of gold layer on polymers or silicon nanotemplates and the removal of the top layer of gold together with the template. Such gold nanobowls are characterized by very broad plasmonic bands due to the plasmon hybridization. The sharp edges on the top of nanobowls are potential sources of the strong electromagnetic field beneficial for SERS. We present a novel template-free synthesis of gold nanobowls (AuNBs). The AuNB layers are deposited on graphene oxide (GO) layers. We compare AuNBs with gold nanospheres (AuNSs) and gold nanourchins (AuNUs) having similar size. The gold nanoparticles are combined with pristine GO or graphene oxide conditioned in ammonia (GONH_3_) or graphene oxide conditioned in sodium hydroxide (GONaOH). The SERS properties of the hybrid supports were studied using rhodamine 6G (R6G) as the SERS probe. The 633 nm laser line was used, which falls out of the molecular resonance with R6G. The results indicate that AuNBs show largely higher enhancement factors when compared to AuNUs and AuNSs. Furthermore, the GO materials are able to modify the SERS enhancement by 1 order of magnitude. We explain the influence of the GO material by three factors: (1) enabling or disabling the charge transfer between gold and R6G, which is crucial for the chemical part of SERS enhancement; (2) causing the aggregation of gold nanoparticles and formation of hot spots; (3) dipole contribution to the electromagnetic enhancement through the abundance of polar groups on the surface.

## Introduction

The effect of shape of gold nanoparticles on their properties is widely studied because of their theranostic,^1,2^ catalytic, and wide range of analytical applications.^3,4^ Among analytical methods, surface-enhanced Raman spectroscopy (SERS) acquires broad attention due to the fingerprint-like signal from the Raman spectrum and exceptional sensitivity, which enables obtaining signals from very low concentrations of the analyte or even single molecules.^5,6^ The major factor causing the enhancement of the Raman signal is the intense electromagnetic field generated by the resonant plasmonic excitations upon laser light shining on noble metal nanostructures. The type of the metal and shape of the nanoparticles determines the plasmonic properties.^7,8^ Several reports show that protrusions, sharp edges, spines, and nanogaps between nanoparticles give rise to the strong local electromagnetic field magnitudes called hot spots,^9^ busting the intensity of SERS spectra. When the SERS support involves graphene or semiconductive materials, the local electromagnetic field might be enhanced by dipoles induced by the incident laser beam. Such enhancement is called dipole mechanism.^10^

The chemical coupling between the SERS active support and the adsorbed molecule enables charge transfer transitions between the support and the molecule. When the energy of such transition falls into the resonance with the exciting laser beam, the Raman signal is enhanced in a similar way as the resonant Raman spectra.^11^ Such chemical enhancement can work in parallel with the electromagnetic one. In early SERS studies of rough electrodes, it was observed that the SERS intensity depends on the electrode potential, which was rationalized by the chemical effect. The Fermi level of the electrode metal shifts with the change of electrode potential, and this shift changes the resonance condition and the Raman intensity in turn.^12^

Gold nanobowls (AuNBs) are potentially attractive as SERS substrates because they have sharp edges on one side, where an intense electromagnetic field can be formed. They are characterized by broad plasmonic bands due to the mixing of the possible plasmonic excitations inside the cavity and outside of the nanobowl. Ye et al. prepared gold nanobowls by using polystyrene nanoparticles as templates. The authors covered the template with a layer of gold, which was subsequently removed by the ion milling technique, and a vapor HF etching technique was used to remove the polystyrene template. It was shown experimentally that such AuNBs gave broad plasmonic bands.^13^ Chen et al. used silica templates to obtain the AuNB arrays oriented with the bowl opening up or down. Intense SERS spectra are observed, if the open part is oriented to the top, thus exposing the sharp metal edges.^14^ Sun et al. used the nanopolystyrene template to obtain silver nanobowls and O_2_-etched polystyrene covered by a silver layer to form nanohole-like arrays. The nanobowls exhibit stronger SERS signals than nanohole-like arrays. The observation has been explained by the effect of the gaps between adjacent nanobowls and sharp edges giving a strong SERS enhancement.^15^ There are no reports on chemically template-free synthesized silver or gold nanobowls applicable for SERS up to our knowledge.

In this work, we propose the novel template-free synthesis of AuNBs. We compare the AuNBs with gold nanourchins (AuNUs) and gold nanospheres (AuNSs). AuNUs are characterized by the high surface area available for the interaction with adsorbing species^16^ and intense electromagnetic fields at the top and between the spines.^17^ AuNSs have been used as SERS supports for a long time,^18^ being a good standard to compare the properties of SERS platforms.

Combining noble metal nanoparticles with graphene materials brings new benefits like improved photostability and quenching of fluorescence of the analyte molecules, widening the applications of SERS.^19^ Graphene enhances the Raman signal from adsorbed molecules via the chemical effect and quenches their fluorescence.^20^ The Raman spectrum of graphene consists with only few well-defined bands, which do not obscure the interpretation of spectra of the adsorbed species. Graphene oxide, (GO), contrary to graphene, has numerous defects and surface groups, which broaden its Raman bands, but give the fascinating chemical flexibility of GO. Reducing GO changes its electric conductivity due to the partially restored sp^2^ carbon–graphene-like domains. Furthermore, the reduction affects the hydrophilic–hydrophobic characteristics, which influence the adsorption of organic molecules in turn. Materials combining gold nanoparticles and GO or reduced graphene oxide (RGO) show exceptional physicochemical properties. Very often, the synergy between gold nanoparticles and RGO leads to enhanced electrocatalytic properties.^21^ The nanoparticle–GO hybrid materials have been applied in electrochemical sensors^22,23^ or supercapacitors.^24^ Encapsulation of gold nanoparticles in GO reduces their cytotoxicity, expanding the possible bioapplications of gold nanoparticles.^25^ Studies of the interactions between GO materials and noble metal nanoparticles are thus interesting for quite a broad application range. The influence of GO on the SERS properties of hybrid materials is still a matter of discussion.^26,27^ The factors influencing the SERS efficiency are the number of GO layers,^28^ the degree of reduction, and/or the oxygen surface groups.^10,27,29,30^

Recently, we have shown that a simple room-temperature treatment of GO in ammonia solution significantly improves the SERS efficiency of composites of AuNSs or AuNUs with graphene oxide.^26^ Such treatment causes a partial reduction of GO by removing the epoxide functional groups. It also causes an abundant adsorption of ammonia ions on the GO surface. The adsorbed ions contribute to SERS by the dipole mechanism presumably.

In this article, we present the template-free synthesis of AuNBs. We combine AuNBs with pristine GO and GO conditioned in ammonia solution (GONH_3_) or GO conditioned in sodium hydroxide solution (GONaOH). The hybrid materials are tested as possible SERS platforms using rhodamine 6G (R6G) as the molecular probe. We compare AuNBs with AuNSs and AuNUs. All materials are characterized by SEM, TEM, UV–vis, infrared, and Raman spectroscopy analyses .

## Methods

### Chemicals and Reagents

All reagents were available commercially and were used as received. Tetrachloroauric acid (trihydrate, HAuCl_4_·3H_2_O, >99.9%), silver nitrate (AgNO_3_, 99.9999%), sodium citrate dihydrate, HOC(COONa)(CH_2_COONa)_2_·2H_2_O (>99.9%), sodium borohydride (powder, NaBH_4_, 98%), and rhodamine 6G (R6G, 99%) were purchased from Merck. Nitrogen gas (purity 99.999%) was purchased from Air Products (Poland). Sodium hydroxide (NaOH) was purchased from Avantor Performance Materials (Poland). Hydrogen peroxide (H_2_O_2_, 30%) and ammonia solution (NH_4_OH, 25%) were purchased from Chempur (Poland). Potassium bromide (KBr, spectroscopic grade) was purchased from Pike Technologies. All solutions were prepared using distilled water (Milli-Q, 18.2 MΩ•cm). Solutions for nanoparticle synthesis were deoxygenated by bubbling with nitrogen gas.

### Synthesis and Conditioning of GO

GO was synthesized using our modified Hummers–Offeman method.^26,31^ Briefly, 6 g of graphite powder was added into 150 mL of concentrated sulfuric acid. The ingredients were mixed while being cooled in an ice bath to avoid overheating. Subsequently, 21 g of potassium permanganate was added under stirring (slowly, to keep the temperature below 30 °C). After 2 h of stirring, 150 mL of distilled water was added slowly, and then 35 mL of hydrogen peroxide was added. The as-prepared GO was centrifuged at 6000 rpm for 30 min and washed four times with distilled water and left to dry. GO obtained with this procedure evinces a high epoxide group content, important for further modifications. To obtain ammonia-modified GO (GONH_3_), 20 mg of GO was mixed with 3 mL of 3% ammonia solution, sonicated for 30 min, and mixed with a magnetic stirrer overnight. The as-prepared GONH_3_ was centrifuged at 6000 rpm for 30 min, washed with water, and left to dry. The same conditioning procedure was performed again, using 3 mL of 3% solution of NaOH instead, to obtain GONaOH.

### Synthesis of Nanoparticles

AuNSs and AuNUs were prepared using the procedure described in our previous work.^26^ AuNBs were synthesized using the following method: 15 mL of 6 mM solution of sodium citrate was mixed with 3 mL of 4.3 mM solution of HAuCl_4_ and 2 mL of 7.8 mM AgNO_3_. The as-prepared solution was mixed for 3 min under the flow of nitrogen gas. Separately, a solution of 25 mL of 30% H_2_O_2_ and 1.5 mL of 0.03 M NaBH_4_ was prepared and deoxygenated using nitrogen gas. After deoxygenation, the solution was added to the mixture of metal precursors and mixed for 1 min. Subsequently, 15 mL of 6 mM solution of sodium citrate was added to the mixture and stirred for 2 h. AuNBs were then centrifuged and washed three times with distilled water.

### Preparation of SERS Supports

SERS substrates were obtained using a layer-by-layer method on a glass covered with a 70 nm layer of gold. For all types of GO, 0.2 mg/mL solution was made. Next, a thin layer of appropriate GO was adsorbed on the surface of the support by immersing the gold-covered plates in the GO solution for 24 h. Then, 30 μL of the nanoparticle solution was drop-casted on the substrate and was left to dry. To perform the SERS experiment, 40 μL of ethanol solution of rhodamine 6G with various concentrations was drop-casted on the SERS substrate and left to dry before the measurement.

### Instrumentation

The nanoparticles were studied by HRTEM using a Talos F200X, FEI instrument. The applied voltage was equal to 200 kV.

The morphology of the hybrid layers was studied using a Merlin field emission scanning electron microscope (Zeiss, Germany) at an operating voltage of 3 kV. The samples were deposited on ITO glass. The approach was identical to the preparation of SERS-active substrates. ITO glass was used to avoid the interference from the gold-coated glass. The elemental composition of samples was analyzed using an energy-dispersive X-ray fluorescence (EDS) detector at an accelerating voltage of 15 kV. The signal was collected from the area equal to 10 × 15 μm.

Infrared spectra were recorded using a Nicolet iS50 FT-IR spectrometer (Thermo Scientific) with a DTGS detector. The SERS supports were studied by the attenuated total reflection (ATR) spectra using a Smart_iTR accessory (Thermo Scientific) with the single-reflection diamond prism. The sample was pressed against the ATR prism. The ATR accessory exposed to air was used to record the background spectrum. Typically, 32 scans were used for the sample as well as the background spectrum. The ATR correction implemented in the spectrometer-controlling program (OMNIC) was used.

Raman spectra were recorded using a DXR Raman microscope (Thermo Scientific) with 50×/0.50 NA objective. For the spectra of different GO samples, the following experimental parameters were used: exposure time was 10 s, and six scans were collected. For SERS measurements, the exposure time was 8 s for AuNSs@GO, AuNSs@GONH_3,_ and AuNSs@GONaOH and 1 s for the remaining samples. Typically, six scans were collected. To ensure repeatability, 10 spectra were collected at random spots on the sample and averaged for each concentration of R6G. For each experiment, red laser (633 nm) was used as the source of excitation.

UV–vis absorption spectra were studied using a PerkinElmer Lambda 650 spectrometer. All samples were measured in a quartz cuvette with an optical length of 10 mm.

## Results and Discussion

### Microscopic Characterization

**Figure 1:**
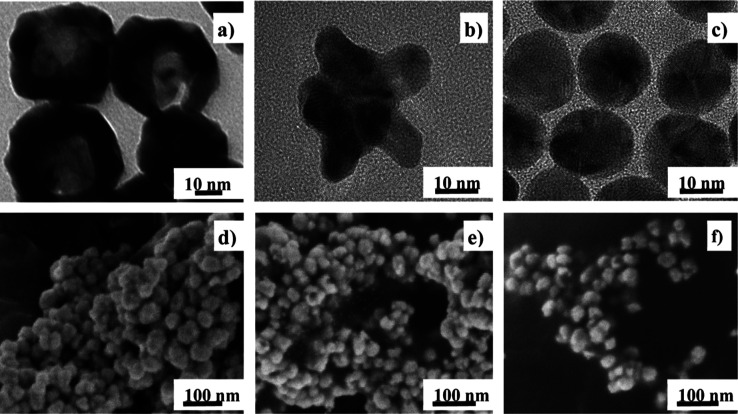
TEM images of AuNBs (a); AuNUs (b); AuNSs (c); and SEM images of AuNBs@GO (d), AuNBs@GONH3 (e), and AuNBs@GONaOH (f).

HRTEM was used to confirm the shape of the studied nanoparticles (Figure 1). The AuNBs are spherical with visible empty interiors (Figure 1a). The AuNUs consist of a core with spines going out of it (Figure 1b). The AuNSs are plain spheres (Figure 1c). All nanoparticles have similar size varying from 30 to 40 nm. On a flat surface, the nanobowls can take two different orientations: one with the opening oriented on the top and the opposite one with its cavity directed to the bottom of the surface. To verify the orientation of AuNBs, we studied their SEM images on GO (Figure 1d), GONH_3_ (Figure 1e), and GONaOH (Figure 1f). The SEM results indicate that AuNBs tend to orient with the opening toward the surface in the case of all types of GO. AuNBs also show a tendency to aggregate. The small distances between AuNBs may result in the formation of hot spots. The pictures of AuNBs@GO, AuNBs@GONH_3_, and AuNBs@GONaOH are very similar; thus, ordering of AuNBs on the surface does not depend on the pretreatment of GO in the case of AuNBs.

Similar results are obtained for AuNUs at the three types of GO (Figure S1 in Supporting Information); thus, aggregation of nanoparticles is unlikely a reason for the SERS properties between AuNUs and AuNBs. Among the studied supports, only AuNSs@GONaOH looks different in the SEM pictures (Figure S2). The nanoparticles are rather isolated from each other, suggesting that AuNSs@GONaOH might form less hot spots, and its efficiency as a SERS support might be worse when compared to other studied materials because the formation of hot spots is rather improbable. The isolated nanoparticles visible in the SEM of AuNSs@GONaOH stay in agreement with the EDS results, showing that the content of gold equals 0.58 ± 0.20%, 1.02 ± 0.35, and 0.11 ± 0.07% for AuNSs@GO, AuNSs@GONH_3_, and AuNSs@GONaOH respectively. For AuNBs and AuNUs, the amount of gold on various GO is rather similar for all types of GO, and it equals ca. to 0.3–0.4%.

### UV–Vis Absorption of the Nanoparticle Hybrid Materials

**Figure 2:**
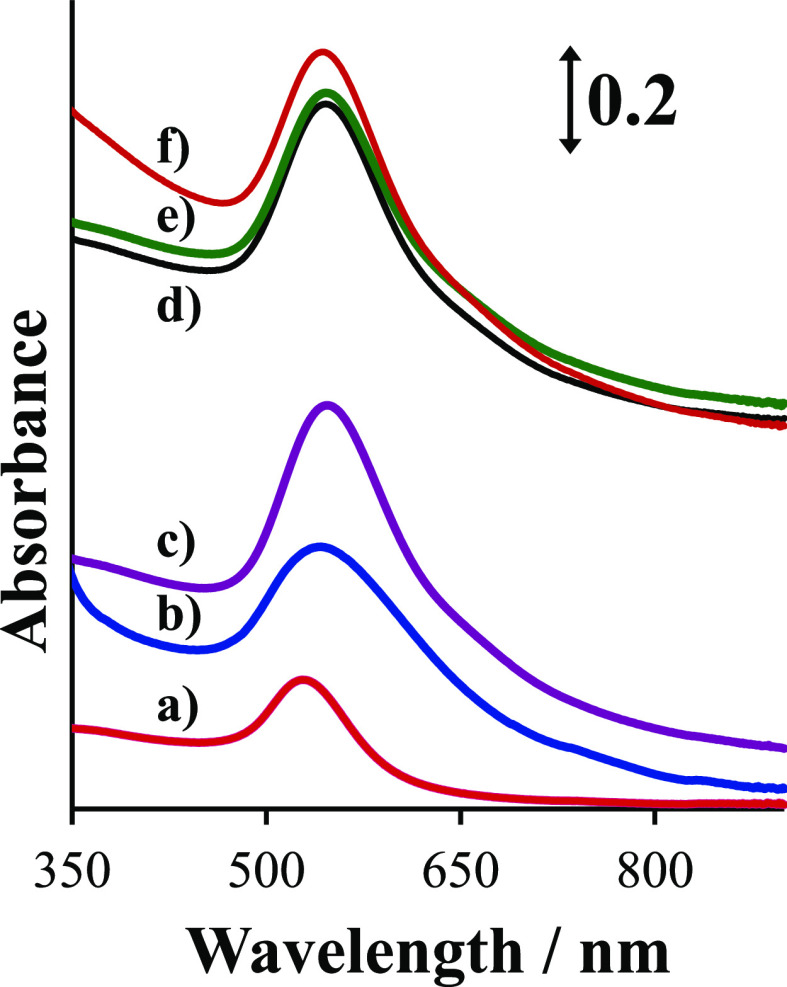
UV–vis absorption spectra of AuNSs (a); AuNUs (b); AuNBs (c); AuNBs mixed with GO (d); AuNBs mixed with GONH_3_ (e); and AuNBs mixed with GONaOH (f).

The efficient SERS requires that the excitation laser wavelength falls into the plasmonic absorption range of the support. The plasmonic properties of gold nanoparticles were characterized using UV–vis spectroscopy. AuNBs give a single band with a maximum at 547 nm, as illustrated in Figure 2. The plasmonic band is quite narrow, indicating rather a homogeneous size of the chemically prepared AuNBs. There is no literature data presenting the expected position of the AuNB plasmonic band in a sol form, nonetheless AuNBs obtained on solid substrates show a broad absorption that varies in the 550–700 nm range.^13^

AuNUs present a similar spectrum, but the maximum is slightly red-shifted to 545 nm and broader, probably due to the irregular shapes of AuNUs. AuNSs present a single band, with the maximum at 531 nm.

To verify whether the interaction with GO materials influences the plasmonic properties of the studied gold nanoparticles, we prepared suspensions of GO materials mixed with gold nanoparticles in water. The spectra of AuNBs mixed with GO, GONH_3_, and GONaOH are shown in Figure 2d–f. The absorption maxima for AuNBs mixed with GO and AuNBs mixed with GONH_3_ occur at exactly the same position as for the plain AuNB suspension, suggesting that the interaction with GO or GONH_3_ does not influence the plasmonic properties of AuNBs. For AuNBs mixed with GONaOH, the position of the maximum is slightly blue-shifted to 542 nm. Furthermore, the spectral background is higher––especially in the low-wavenumber part of the spectrum (Figure 2f), suggesting that GONaOH interacts strongly with AuNBs. The electronic transitions, which contribute possibly to such a background, are related to GO π → π* of C=C and *n* → π* C–O,^32^ and transitions of electrons from the occupied d-level states to the empty states above the Fermi level of gold appear in a similar spectral range.^33^

Comparison of the UV–vis spectra of plain AuNUs and AuNSs to their mixtures with GO materials leads to similar results (Figures S3 and S4 in Supporting Information). Generally, the interaction of GO materials with gold nanoparticles has rather little effect on the shape of the plasmonic band. However, there are some differences in the spectral background, suggesting that the interaction between the nanoparticles and GO materials causes other effects, which are not related to plasmonic absorption but rather the properties of GO.

### Infrared Spectra of AuNBs, AuNSs, and AuNUs and Their Composites with GO Materials

The most intensive bands in the infrared spectra of all studied nanoparticles correspond to the asymmetric (ν_as_) and symmetric (ν_s_) stretching modes of the dissociated carboxylic groups originating from the citrate ligands stabilizing nanoparticles (Figure S5). The ν_as_ band is positioned at 1584, 1579, and 1581 cm^–1^ for AuNSs, AuNUs, and AuNBs respectively. The ν_s_ band appears at 1399, 1396, and 1395 cm^–1^. The position of the COO^–^ modes and the distance between ν_as_ and ν_s_ are sensitive to the binding of the carboxylic group to the gold surface.^34^ Very close band positions observed for the three types of nanoparticles suggest that the binding of the carboxylic groups is similar. The observed values suggest that the COO^–^ bridge conformation dominates.^34,35^

The spectra of all studied nanoparticles show broad OH stretching bands ranging between 2500 and 3600 cm^–1^. The spectra of AuNBs and AuNUs show clear components of the OH stretching band at 2800 cm^–1^, while in the spectra of AuNSs, it cannot be distinguished clearly. Such a low frequency of the OH stretching mode signifies the presence of strong hydrogen bonds, as, for example, in the dimers of carboxylic acids.^36^ The band at 2800 cm^–1^ might originate from the dangling citrates, which are attached by hydrogen bonds to the ligands directly interacting with the gold surface.^35^ A relatively high intensity of this band in the case of AuNBs and AuNUs results probably from the large surface area of these nanoparticles and the abundance of citric ligands.

**Figure 3:**
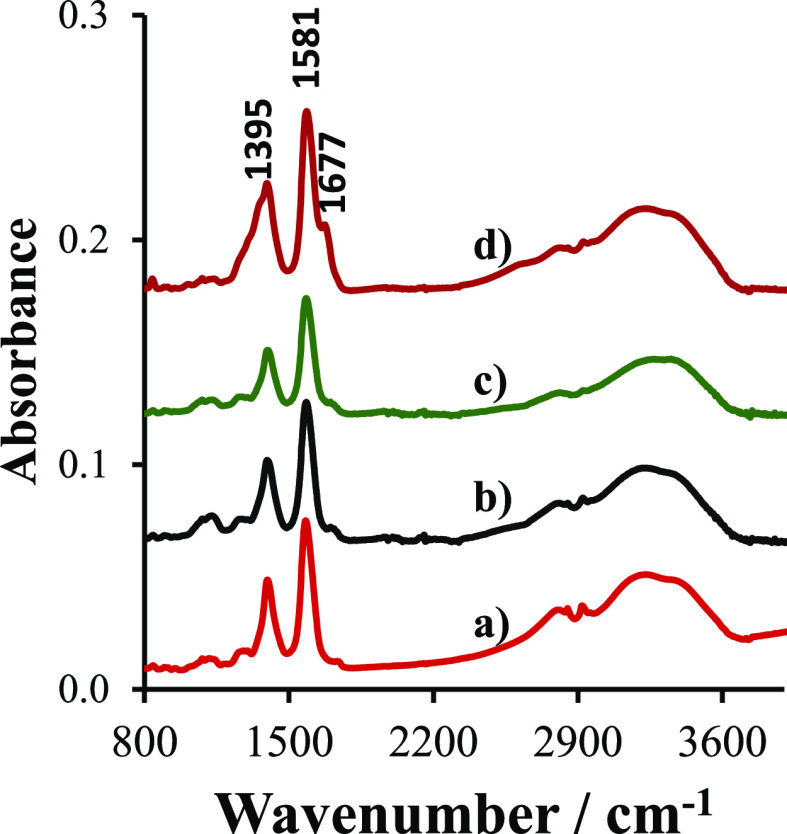
Infrared spectra of AuNBs (a); AuNBs@GO (b); AuNBs@GONH_3_ (c); and AuNBs@GONaOH (d).

Previous studies showed that the conditioning of GO in ammonia or NaOH solution causes the opening of the epoxy rings and the adsorption of ammonia ions, which are visualized in the infrared spectra by the diminishing of the characteristic bands of the epoxy groups at 1225 cm^–1^ and the increase of the band at ca. 1400 cm^–1^ due to the diol OH bending or NH bending (in the case of GONH_3_).^26^ Besides the epoxide groups, GO materials contain carboxylic and phenol groups, which contribute to the infrared spectrum. Comparing the spectra of AuNBs , AuNBs@GO, and AuNBs@GONH_3_ (Figure 3a–c), it is visible that the addition of GO causes only a minor change in the spectrum, which can be rationalized by the fact that COO– and OH groups are present in both AuNBs and GO. Another possible reason for the large similarity of the spectra is the SEIRA effect,^37^ causing an enhancement of the bands due to the groups directly interacting with the gold surface, which are citrate ligands. However, in the spectrum of AuNBs@GONaOH (Figure 3d), changes are noticeable. The shoulder above 1700 cm^–1^, originating from the COOH groups, is replaced by the band at 1677 cm^–1^, corresponding to the COO– asymmetric stretching mode. The symmetric stretching band at 1395 cm^–1^ becomes broader. Such changes suggest that the COOH groups in the coordination sphere of AuNBs dissociate in contact with GONaOH.

In summary, the infrared spectra indicate that AuNBs interact differently with GONaOH than with GO or GONH_3_. The interaction causes the dissociation of the carboxylic groups of citrate ligands coordinated to gold, and it changes the conformation of the citrate ligands at the surface.

For comparison, we studied the spectra of AuNUs and AuNSs mixed with GO, GONH_3_, and GONaOH (Figures S6 and S7 in Supporting Information). The results are similar to those described for AuNBs. Thus, the contact of AuNUs or AuNSs with GONaOH causes the dissociation of all COO– groups.

Generally, infrared results indicate that the interaction of AuNBs, AuNUs, or AuNSs with GO materials causes mainly the dissociation of COOH groups and possibly also the change of the conformation of citrate ligands. Such changes might indirectly affect the SERS properties of the nanoparticles because the dissociation influences the charge of citrate ligands. The charge influences, in turn, the Fermi level of gold, which is important for the chemical part of the SERS enhancement.

### AuNBs, AuNUs, AuNSs Composites with GO Materials—Raman spectra

**Figure 4:**
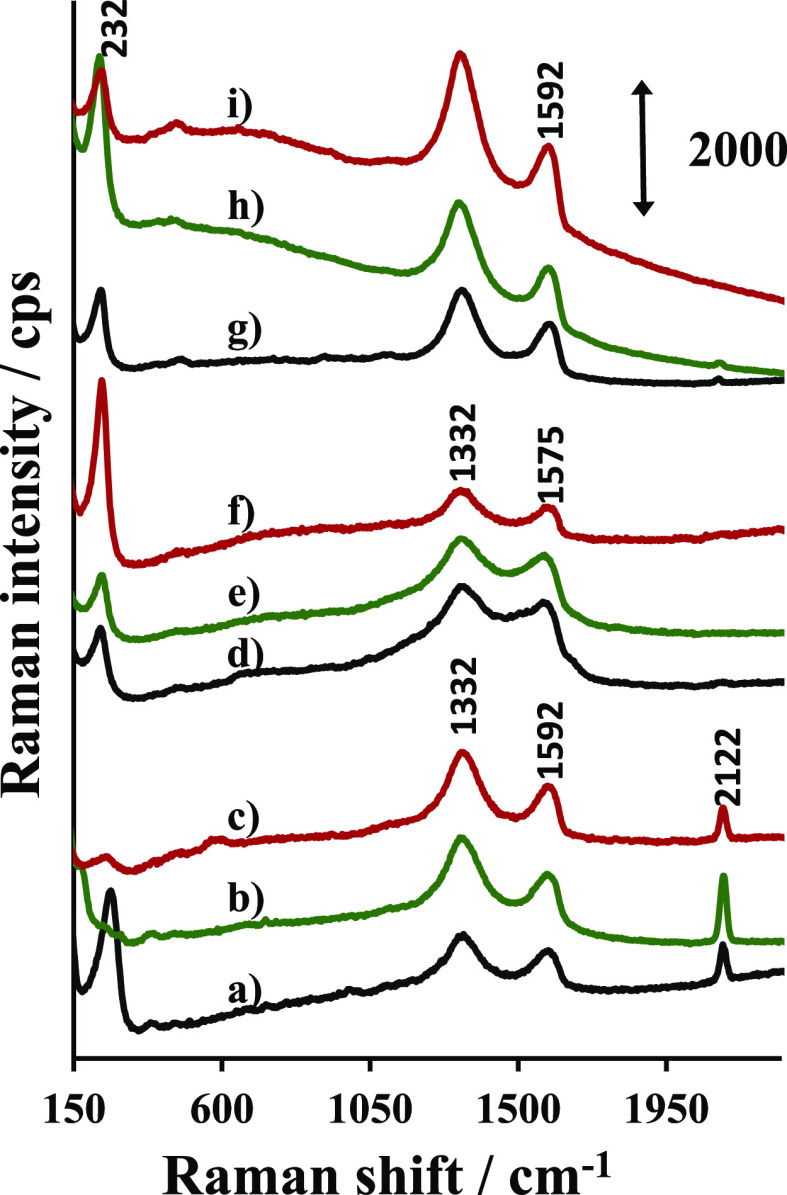
Raman spectra of AuNSs@GO (a); AuNSs@GONH_3_ (b); AuNSs@GONaOH (c); AuNUs@GO (d); AuNUs@GONH_3_ (e); AuNUs@GONaOH (f) AuNBs@GO (g); AuNBs@GONH_3_ (h); and AuNBs@GONaOH (i).

Raman spectroscopy is an excellent tool for studying graphene family materials as it is sensitive to the defects and surface functionalization of the graphene plane.^38^Figure 4 compares the Raman spectra of the three types of gold nanoparticles studied here combined with different types of GO. All spectra show the D and G bands characteristic for graphene materials, though different relative intensities and shifts in band positions are noticeable. The G band is observed at 1592 cm^–1^ for the composites of AuNSs and AuNBs, while for composites involving AuNUs, the G band is red-shifted to 1575 cm^–1^. The position of the G band is sensitive to the content of oxygen; thus, the observed difference in the G band position suggests that the composites of AuNUs with GO materials contain more oxygen surface groups.^39^ The D band position is very similar for all composites and is equal to 1332 cm^–1^. The spectra of AuNUs@GO, AuNUs@GONH_3_, and AuNUs@GONaOH show very broad—nearly overlapping––bands in general. Such spectra indicate very defective structures, which result probably from the strong interaction between AuNUs and GO materials, leading to their oxidation and fragmentation.

The D-to-G intensity ratio is a popular parameter to estimate the amount and the size of defects. According to the Tuinstra–Koeing model, the *I*_D_/*I*_G_ ratio is inversely proportional to the size of crystallites in carbon materials.^40^Table 1 presents the *I*_D_/*I*_G_ values for the studied composites and for the layers of GO, GONH_3_, and GO NaOH prior to the deposition of the nanoparticles. If all values in the rows of Table 1 are identical, the defects are independent of the interaction with GO. However, these values are not equal, so just the mixing of the nanoparticles with GO influences the structure of GO. As visible, both the conditioning of GO in a basic solution and the interaction with gold nanoparticles cause the formation of defects on GO planes. Comparing the *I*_D_/*I*_G_ values for the three types of GO, the amount of defects can be ordered as follows: GO < GONH_3_ < GONaOH. Comparison of the values in the rows of Table 1 suggests that the interaction between gold nanoparticles and GO materials gets stronger in the order: AuNSs < AuNUs < AuNBs. This last order suggests that AuNBs might be more sensitive to the interaction with GO than AuNUs and AuNSs.

**Table 1:** *I*_D_/*I*_G_ Values of the Studied Composites

	*I*_D_/*I*_G_		*I*_D_/*I*_G_		*I*_D_/*I*_G_		*I*_D_/*I*_G_
AuNBs@GO	1.57	AuNUs@GO	1.28	AuNSs@GO	1.17	GO	1.20
AuNBs@GONH_3_	1.67	AuNUs@GONH_3_	1.38	AuNSs@GONH_3_	1.44	GONH_3_	1.32
AuNBs@GONaOH	1.86	AuNUs@GONaOH	1.58	AuNSs@GONaOH	1.48	GONaOH	1.42

Besides the bands due to GO, the spectra of the composites show low-frequency bands, which can be attributed to the Au–Cl stretching mode. The chloride ions originate from HAuCl_4_, which is used as the gold precursor. The position of the Au–Cl band ranges from 232 cm^–1^ in AuNBs@GO and AuNUs@GO to 262 cm^–1^ for AuNSs@GO. Such shifts of the band position suggest that the force constant of the Au–Cl bond is affected by the shape of the gold nanoparticles.

Another band which cannot be assigned to GO occurs at 2122 cm^–1^ as it is observed in the spectra of bare AuNSs (not shown). It is the strongest for AuNSs@GO (Figure 4a). There are only few chemical species, which give a response in such a wavenumber range, for example, CO chemisorbed on Au(110)^41^ or carbonyl metal complexes.^36^ We suppose that the band near 2100 cm^–1^ indicates the presence of carbonyl groups strongly interacting with gold. The band is rather narrow, suggesting that there are citrate ligands interacting solely by one carbonyl group with the gold surface. AuNSs contain probably more of such ligands compared to AuNUs and AuNBs.

The observed differences suggest that the shape of the gold nanoparticle influences the Raman spectra more than the type of GO material, as the *I*_D_/*I*_G_ values are the highest for all AuNB composites, the position of G band is lower for AuNUs—not depending on the type of GO––and the band near 2100 cm^–1^ is the highest for AuNSs. The interaction between gold nanoparticles and GO thus depends on the shape of the nanoparticle, so the effect of GO on the SERS properties of gold nanoparticles could be different for AuNBs, AuNUs, and AuNSs.

### SERS Spectra of R6G

**Figure 5:**
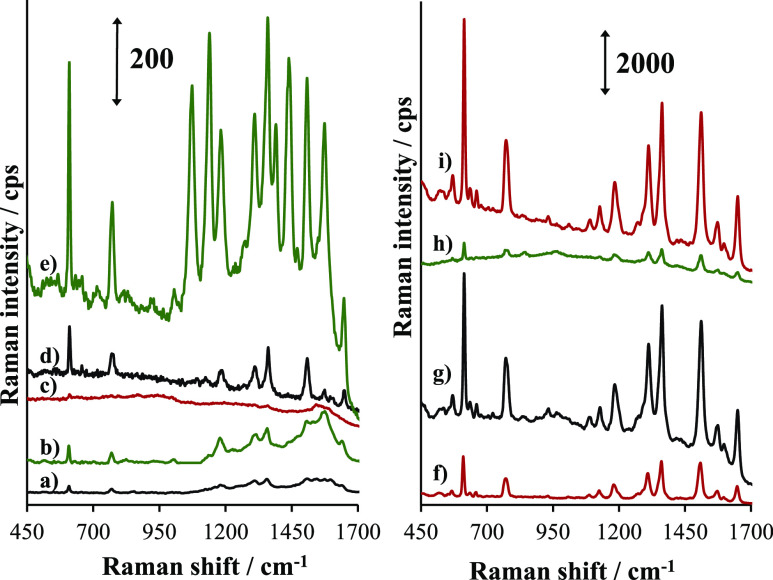
SERS spectra of 10^–6^ M R6G on AuNSs@GO (a); AuNSs@GONH_3_ (b); AuNSs@GONaOH (c); AuNUs@GO (d); AuNUs@GONH_3_ (e); AuNUs@GONaOH (f); AuNBs@GO (g); AuNBs@GONH_3_ (h); and AuNBs@GONaOH (i). The intensity scale is different for the plots on the left and right. Arrows indicate the Raman intensity unit.

In order to illustrate the differences in the SERS performance of the studied supports, the spectra of 10^–6^ M R6G are compared in Figure 5. The spectra are highly homogeneous within the studied samples. The standard deviation of the 613 cm^–1^ peak height was below 5% for all the studied supports. Such a value stays well below the accepted standard deviations reported in SERS studies.^42^ The intensity of the SERS spectra depends significantly on the shape of gold nanoparticles. The weakest intensities are observed for AuNSs (plots a, b, and c in Figure 5), and moderate intensity is observed for AuNUs (plots c, d, and f). The spectra obtained for AuNBs are the strongest (Figure 5g–i). Due to the large differences in intensities, the spectra were divided into two plots with different ordinate scales.

In addition to significant differences in enhancement, there are clear differences in the relative intensities of the bands. For example, the bands at 1073, 1128, 1439, and 1574 cm^–1^ are stronger in the spectrum of AuNUs@GONH_3_ (Figure 5e) compared to that in AuNUs@GO (Figure 5d) or AuNUs@GONaOH (Figure 5f). The band at 1574 cm^–1^ is relatively strong also for AuNSs@GO (Figure 5a) and AuNSs@GONH_3_ (Figure 5b).

**Figure 6:**
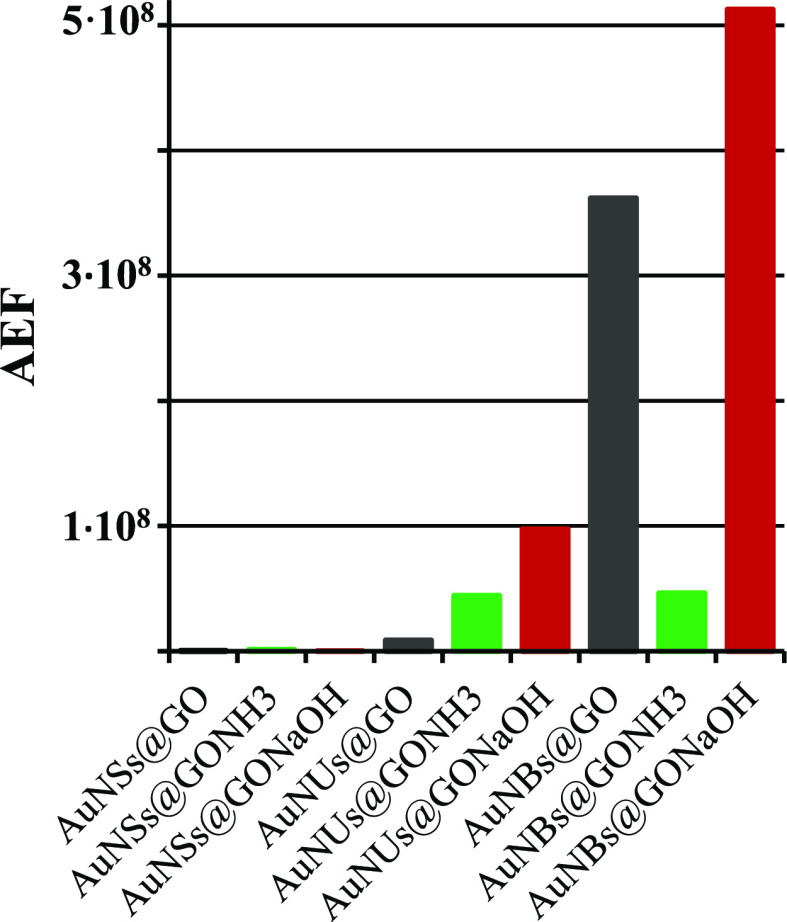
Comparison of the enhancement factors of the studied SERS supports—calculated for the 613 cm^–1^ band.

To get the quantitative comparison of the studied supports, we calculated the analytical enhancement factor (AEF).^42,43^ The enhancement factor was calculated using the following formula
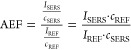
where *I*_SERS_ is the intensity of the band in the SERS spectrum, *C*_SERS_ is the concentration of R6G, and *I*_REF_ is the intensity of the reference Raman spectrum. We used the spectrum of the R6G layer, which was prepared by drop-casting 0.1 M R6G on a microscope glass as the reference. *C*_REF_ is the concentration of the drop-casted R6G solution. We have chosen the band at 613 cm^–1^ as it does not overlap with the bands of GO. The calculated values are collected in Table S1. Figure 6 compares the obtained values in the bar graph.

We repeated the calculation using the spectrum of 0.001 R6G on the glass covered with 70 nm of gold in order to estimate the influence of the underlying gold onto the AEF value. The resulting values are ca. two orders of magnitude lower (see Table S1), meaning the underlying gold layer affects the enhancement, although the order of AEFs remains the same and the AuNBs@GONaOH composite performs significantly better than other supports.

As visible, the enhancement factors obtained for AuNBs are largely higher compared to AuNSs and AuNUs in general, indicating that AuNBs enhance the Raman spectra very efficiently. However, the type of support is very important too. The AEF value obtained for AuNBs@GONaOH is the highest and is equal to 5.13 × 10^8^. The limit of detection (LOD) can be calculated as 3 times value of the noise of the blank spectrum divided by the value of the slope of the calibration plot.^44^ Such estimation gives LOD values in the nanomolar range for the supports involving AuNBs and about 10 or 100 times worse values for AuNUs and AuNSs, respectively (Table S1).

The values of AEF for the AuNB supports are similar to those reported for cubic gold nanoparticles^44^ and slightly higher compared to that of gold nanoparticle assemblies.^45^ The latter are similar to AuNSs on GO supports. Higher AEF values are reported, if the excitation laser line falls in resonance with the R6G absorption. For such surface-enhanced resonance Raman systems,^46,47^ AEF values of 10^11^–10^12^ are possible.

All supports studied here show high stability, if stored in the room temperature after fabrication. After 6 months, about 80–90% of the initial SERS signal intensity was observed.

The SERS performance of AuNBs is better compared to that of AuNUs, which are considered to be very good SERS supports,^16,17^ making them excellent candidates for SERS applications. The large influence of the shape of the gold nanoparticles on the SERS intensity confirms that the electromagnetic factor is the most important for the SERS enhancement, though the enhancement depends clearly on the type of GO material on which gold nanoparticles are deposited. For example, the enhancement factor for AuNBs@GO is ca. 3 times higher than for AuNBsGONH_3_ and about 1 time lower compared to that for AuNBs@GONaOH. It is also noticeable that the influence of GO is not straightforward, as the deposition of AuNBs on GONaOH increases the enhancement factor, and the deposition of AuNSs on GONaOH diminishes the SERS signal instead. The UV–vis studies described above indicated that the plasmonic band is not affected by the interaction with GO materials; thus, electromagnetic enhancement is rather not influenced by GO directly. The conditioning of GO in NH_3_ or NaOH influences the aggregation as visible in the SEM micrographs of the studied materials. However, it may influence the electromagnetic enhancement indirectly by causing the aggregation of gold nanoparticles, which may in turn generate hot spots. The change of the surface composition of GO induced by the conditioning in NH_3_ or NaOH can influence the dipole part of the electromagnetic enhancement.^10^

Another mechanism of the SERS enhancement is the chemical factor, which requires the charge transfer between the Fermi level of gold nanoparticles and the R6G fall in resonance with the excitation laser line. We suppose that the conditioning of GO in NH_3_ or NaOH influences the interaction of GO with gold nanoparticles and the resonance condition in turn. Raman spectra of the composites studied here indicate that the interaction between gold nanoparticles and GO materials causes the formation of new defects and/or oxidation of GO. Additionally, the infrared spectra suggest that GO, GONH_3_, and GONaOH differ by the amount of epoxides and by the dissociation state of carboxylic groups. Such differences influence the charge around gold nanoparticles, which affects the Fermi level of nanoparticles in turn. The chemical part of the SERS enhancement is thus affected by the pretreatment of GO in NH_3_ or NaOH before the adsorption of the nanoparticles. The shifting of the Fermi level changes the resonance condition for the charge transfer between the gold nanoparticle and the adsorbed molecule. Such a mechanism explains why the influence of the GO pretreatment is so significant and different for different types of nanoparticles.

Table 2 collects the band positions of R6G observed here together with the band assignments based on refss (48, 49). In the case of all AuNB–GO combinations, the band positions of R6G are similar, with differences being smaller than the resolution of the measurement. The relative intensities do not depend on the type of GO as well, while for AuNUs, the observations are different. The spectrum of 10^–6^ M R6G on AuNUs@GONH_3_ (Figure 5e) has different relative band intensities. The typically strongest bands of R6G at 1508, 1363, 771, and 613 cm^–1^ diminish, while the bands at 1439, 1390, 1128, and 1073 cm^–1^ become very strong. All diminishing bands involve the motion of the xanthene ring (see Table 2), while the increasing bands involve the motions of the NHC_2_H_5_, methyl, and phenyl groups. Such change of the relative intensities suggests that the orientation of the R6G molecules at the surface enables the charge transfer between the NHC_2_H_5_, methyl, and phenyl groups and the support. Such explanation stays in agreement with the proposed significant role of the chemical enhancement for the SERS supports composed of gold nanoparticles and GO. Upon the increase of the R6G concentration, the relative intensities become typical (not shown), thus similar to those observed for AuNBs, suggesting that the molecular orientation becomes random at higher concentrations. An alternative explanation is that at the higher concentration, the contribution of spectra enhanced by the electromagnetic mechanism is more prominent. The CT mechanism is the extra signal enhancement, which operates only for the molecules directly attached to the gold nanoparticle, while the electromagnetic enhancement operates on all molecules within the appropriate distance. When the number of molecules increases, the electromagnetically enhanced spectrum may dominate.

**Table 2:** Band Positions Observed in the R6G Raman Spectra in the Current Work and Assignments Based on Refs (48) and (49)

position ν (cm^–1^)	assignment^a^
313, 358, 404, 451, 522, 570	torsion and/or bend. (rings)
613	X(49), P(48)
771	X(75), A(11), M(12)
842	X(35), A(45), P(11)
1073	A(37), P(57)
1128	X(44), A(32), P(14)
1139	X(21), A(70)
1184	X(82), A(14)
1310	X(30), A(48), P(21)
1363	X(70), A(17)
1390	overlapping contr. from A(94); A(99); M(87); M(95)
	
1439	X(16), A(42), M(42)
1510	X(69), A(25)
1574	P(99)
1596	P(100)
1650	X(98)

a The assignment is based on refs (48) and (49). X refers to the motion of xanthene ring, A refers to the motion of the NHC_2_H_5_ groups, M refers to the methyl groups adjacent to the xanthene ring, and P refers to the phenyl ring with the COOC_2_H_5_ group. The values in brackets are the potential energy distribution of normal modes.

The AuNSs enhance the Raman spectra worse compared to AuNBs and AuNUs. AuNSs@GONaOH is particularly a poor SERS platform as the spectrum of R6G at the concentration of 10^–6^ M is hardly visible. The reason might be the poor adsorption of AuNSs at GONaOH visible in the SEM results, but the chemical effect, which in this case moves the energy of the charge transfer between R6G and the support out of resonance with the laser line, is also possible.

The spectra at AuNSs@GO and AuNSs@GONH_3_ show relative intensities similar to that of AuNUs@GONH_3_, suggesting a similar orientation of the R6G molecule and a significant chemical contribution to the SERS enhancement. Similar to AuNUs@GONH_3_, the increase of the concentration changes the relative intensities, and the spectra resemble those for AuNBs.

## Conclusions

We have successfully fabricated gold nanobowls in a sol form. Combined with various graphene oxides, AuNBs are very efficient SERS substrates, showing higher enhancement factors than AuNUs and AuNSs. GO can modify the SERS enhancement by 1 order of magnitude. The pretreatment of GO in NH_3_ or in NaOH solution changes the SERS enhancement. The influence of GO can be rationalized by the following reasons:

- Chemical part of the SERS enhancement is influenced by the modification of the Fermi level of gold nanoparticles, which in turn modifies the energy of the charge transfer between gold and R6G. Such a shift of the charge transfer energy in or out of resonance with the excitation laser line has possibly a large influence on the SERS intensity, though the effect is not always beneficial.
- Surface groups of GO influence the aggregation of nanoparticles, leading to the formation of hot spots.
- Presence of oxygen surface groups on the GO surface increases the SERS signal through the dipole mechanism.

GO and modified GO materials are promising supports for gold nanoparticles, which can tune the chemical enhancement for very sensitive SERS assays. Due to the resonance character of the chemical part of the SERS effect, the signal enhancement might not be equally efficient for various molecules. Such property could be utilized in future to enhance the selectivity of SERS assays.

## Supporting Information Available

SEM images of AuNUs and AuNSs with various GO materials, UV–vis and infrared spectra of these composites; details of the estimation of the analytical enhancement factors; and limits of detection and values of these parameters for all studied supports (PDF)

## Abbreviations

ATR-FTIR: attenuated total reflection-Fourier transform infrared spectroscopy
AuNBs: gold nanobowls
AuNSs: gold nanospheres
AuNUs: gold nanourchins
CT: charge transfer
GO: graphene oxide
GONaOH: graphene oxide conditioned in NaOH solution
GONH_3_: graphene oxide conditioned in ammonia solution
R6G: rhodamine 6G
SEM: scanning electron microscopy
SERS: surface-enhanced Raman spectroscopy
TEM: transmission electron microscopy
UV–vis: absorption spectroscopy in the ultraviolet and visible range
