# Supplementary material for: Template-Free Synthesized Gold Nanobowls Composed with Graphene Oxide for Ultrasensitive SERS Platforms
Source: J Phys Chem C Nanomater Interfaces. 2023 Aug 18;127(34):16960–9. doi: 10.1021/acs.jpcc.3c03607 (PMC10478765; doi:10.1021/acs.jpcc.3c03607)
Supplement: Supplementary file 1 — jp3c03607_si_001.pdf [file jp3c03607_si_001.pdf]

Template-Free Synthesized Gold Nanobowls Composed with Graphene Oxide for Ultrasensitive SERS Platforms

Mateusz Kasztelan[a,b]*, Sylwia Zoladek*[a]*, Władysław Wieczorek, Barbara Palys*[a]

[a]Faculty of Chemistry, University of Warsaw, Pasteura 1, Warsaw, 02-093, Poland

[b]Faculty of Chemistry, Warsaw University of Technology, Noakowskiego 3, Warsaw, 00-664, Poland

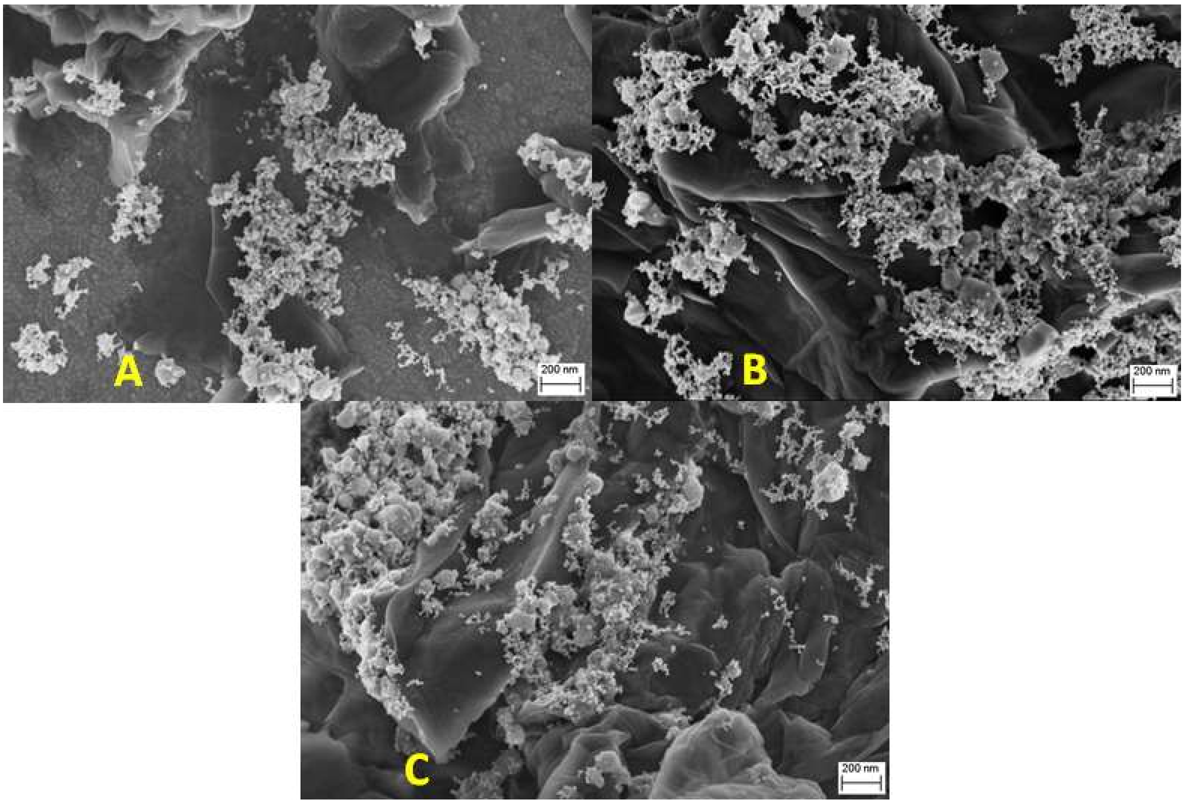

Figure S1. SEM images of AuNUs@GO A), AuNUs@GONH_3 B), AuNUs@GONaOH C).

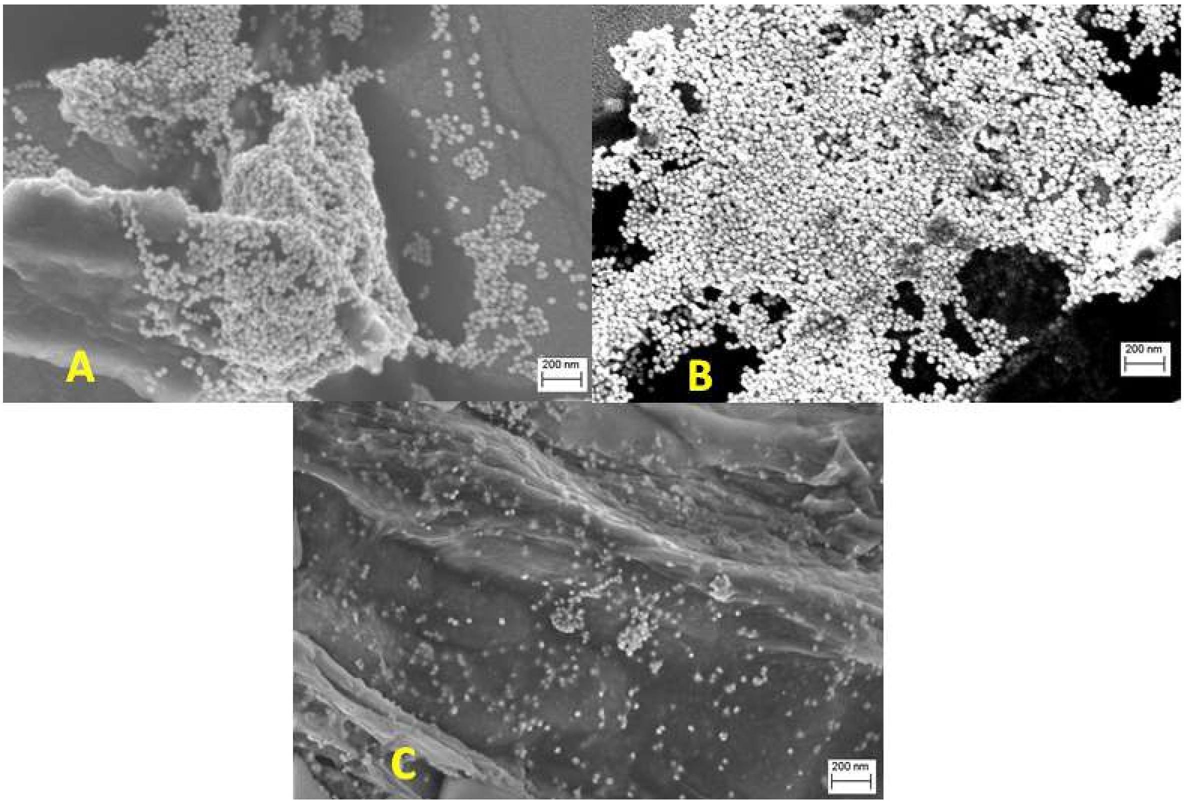

Figure S2. SEM images of AuNSs@GO A), AuNSs@$GONH_3$ B), AuNSs@GONaOH C).

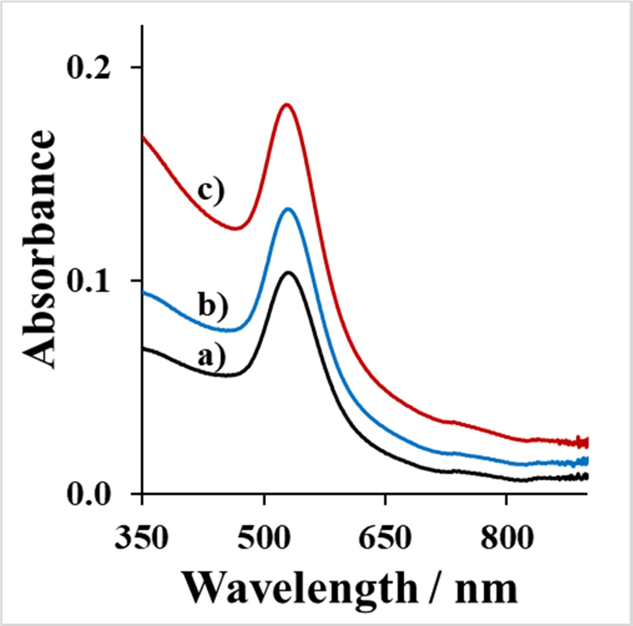

Figure S3. UV-Vis absorption spectra of water suspensions of AuNSs mixed with GO a); $GONH_3$ b); GONaOH.

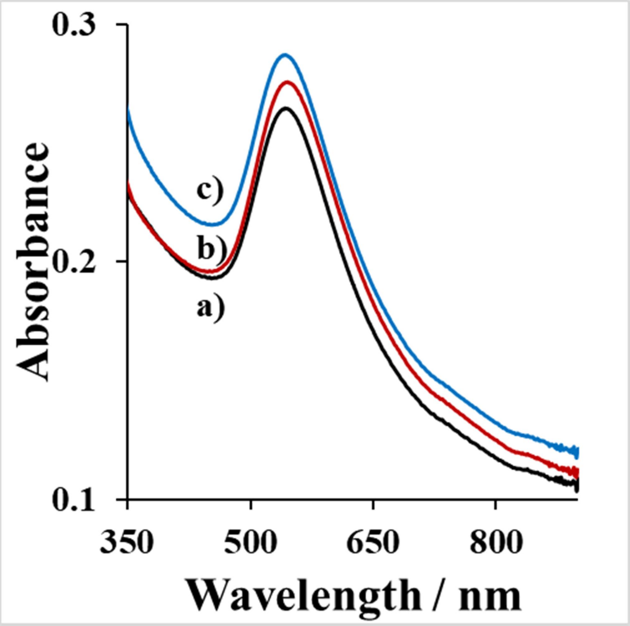

Figure S4. UV-Vis absorption spectra of water suspensions of AuNUs mixed with GO a); $GONH_3$ b); GONaOH c).

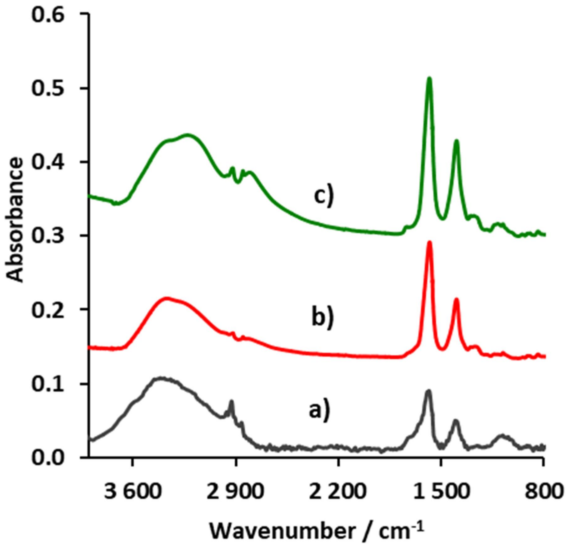

Figure S5. ATR-FTIR spectra of: AuNSs (a); AuNUs (b) and AuNBs (c).

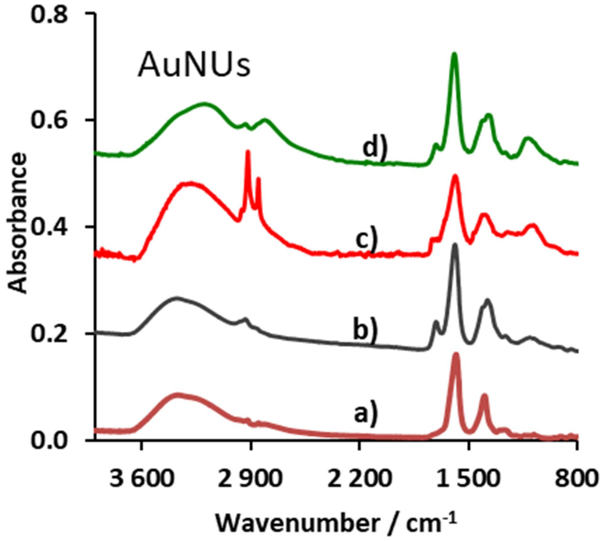

Figure S6. Infrared spectra of AuNUs a); AuNUs@GO b); AuNUs@GONH$_3$ c); AuNUs@GONaOH d).

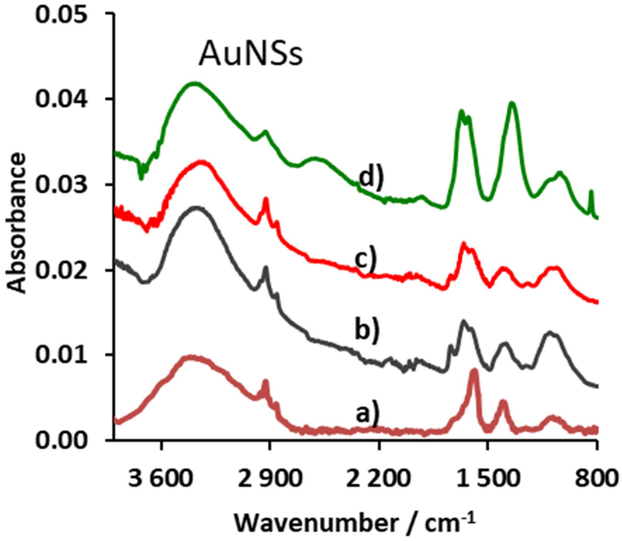

Figure S7. Infrared spectra of AuNSs a), AuNSs@GO b), AuNSs@GONH$_3$ c), AuNSs@GONaOH d).

Estimation of the Analytical Enhancement Factors and Limits of Detection

The Analytical Enhancement Factors (AEF) were evaluated using the following formula:

$$AEF = \frac{I_{SERS}}{C_{SERS}} * \frac{C_{REF}}{I_{REF}} \quad (1)$$

where I_{SERS} and I_{REF} are the intensities of the selected band in the SERS and normal Raman spectra respectively. The C_{SERS} and C_{REF} are the concentrations of the R6G in the SERS and normal Raman experiment. The normal Raman spectrum was recorded for a 0.1 M R6G solution drop-casted on a microscope glass. Both SERS and normal Raman spectra were recorded in exactly the same parameters, thus using the same laser excitation line (633 nm), laser power and the microscope objective (long distance, 50X, N/A 0.5, 1.3 µm laser spot diameter). Typically 1 s exposure time and 4 repetitions were done for a single SERS spectrum. Each spectrum presented in the article is averaged from over 10 individual spectra taken randomly from various points of the support. The exposure times ranged from 1 to 8 s depending on the strength of the signal. The normal Raman spectrum (R6G on glass) required 30 s exposure and 64 repetitions to obtain an acceptable signal to noise ratio.

The studied SERS supports were obtained by the deposition of the appropriate GO solution on the glass covered by 70 nm of gold. The gold nanoparticles were deposited on such layers (see the experimental section in the main text. To estimate the possible effect of the gold layer (underlying GO and the nanoparticles), we have evaluated also the AEF using the R6G drop-casted on gold as the reference spectrum. These values are marked as AEF/Au. As visible, these values are significantly lower, suggesting that the gold layer has influences on the AEF. The values of AEF/Au follow the same pattern as AEF, thus AuNBs are significantly better than AuNUs and AuNSs.

Table S1. Values of the analytical enhancement factors for the band at 613 cm^{-1} of R6G calculated by taking the spectrum of R6G on glass (AEF) or on flat gold (AEF/Au) as the reference. Limit of detection values (3 · noise/slop of the calibration plot).

Support	AEF	AEF/Au	LOD [mol/l]
AuNSs@GO	$4.80 \cdot 10^{5}$	$1.30 \cdot 10^{3}$	$1.84 \cdot 10^{-6}$
AuNSs@GONH$_3$	$1.30 \cdot 10^{6}$	$3.50 \cdot 10^{3}$	$1.17 \cdot 10^{-6}$
AuNSs@GONaOH	$9.95 \cdot 10^{4}$	$3.00 \cdot 10^{3}$	$1.20 \cdot 10^{-6}$
AuNUs@GO	$8.76 \cdot 10^{6}$	$9.50 \cdot 10^{3}$	$6.73 \cdot 10^{-7}$
AuNUs@GONH$_3$	$4.45 \cdot 10^{7}$	$4.56 \cdot 10^{4}$	$1.12 \cdot 10^{-7}$
AuNUs@GONaOH	$9.76 \cdot 10^{7}$	$1.70 \cdot 10^{5}$	$1.66 \cdot 10^{-7}$
AuNBs@GO	$3.62 \cdot 10^{8}$	$4.01 \cdot 10^{5}$	$9.41 \cdot 10^{-9}$
AuNBs@GONH$_3$	$4.46 \cdot 10^{7}$	$5.84 \cdot 10^{4}$	$3.00 \cdot 10^{-8}$
AuNBs@GONaOH	$5.13 \cdot 10^{8}$	$5.28 \cdot 10^{5}$	$6.76 \cdot 10^{-9}$

EDS results for studied supports:

AuNSs@GO – content of gold equal to 0.58 % ± 0.20

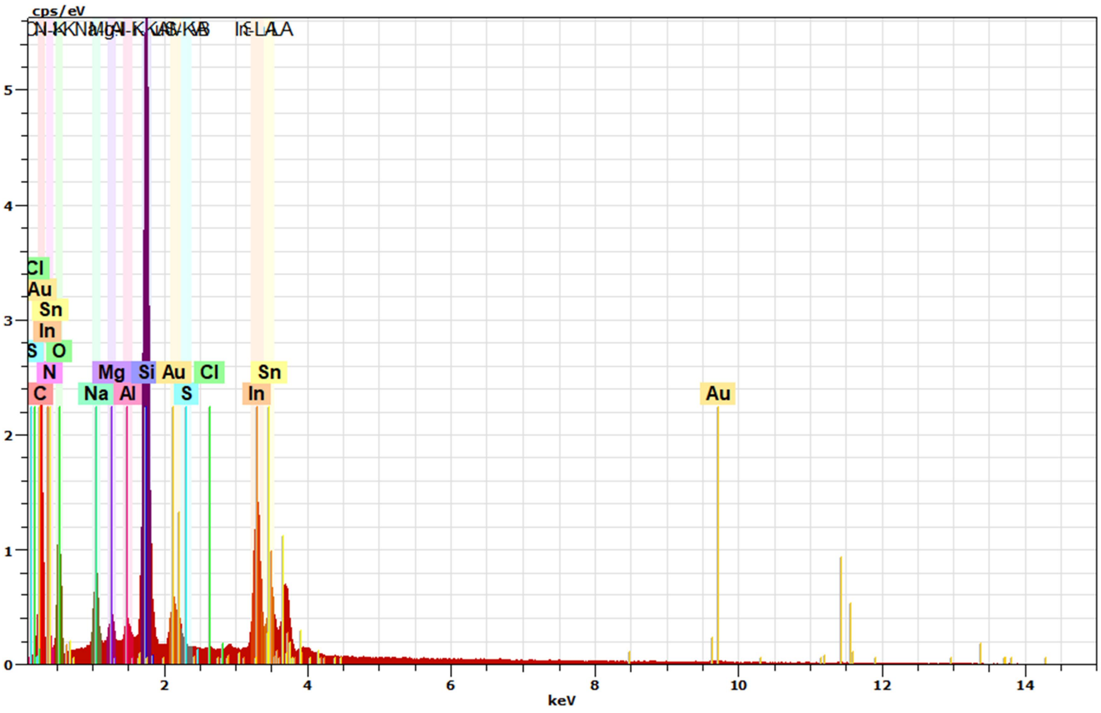

Spectrum: Map

Element	Series	unn. C [wt.%]	norm. C [wt.%]	Atom. C [at.%]	Error (1 Sigma) [wt.%]
Carbon	K-series	25.37	28.35	53.26	3.10
Nitrogen	K-series	0.00	0.00	0.00	0.00
Oxygen	K-series	13.54	15.13	21.34	1.76
Sodium	K-series	2.59	2.90	2.84	0.19
Magnesium	K-series	0.81	0.90	0.84	0.07
Aluminium	K-series	0.54	0.61	0.51	0.05
Silicon	K-series	16.76	18.73	15.05	0.72
Sulfur	K-series	0.07	0.07	0.05	0.03
Indium	L-series	19.63	21.93	4.31	0.62
Tin	L-series	5.65	6.31	1.20	0.20
Gold	M-series	4.52	5.05	0.58	0.20
Chlorine	K-series	0.03	0.03	0.02	0.03
	Total:	89.52	100.00	100.00	

AuNSs@GONH$_3$ – content of gold equal to % 1.02 ± 0.35

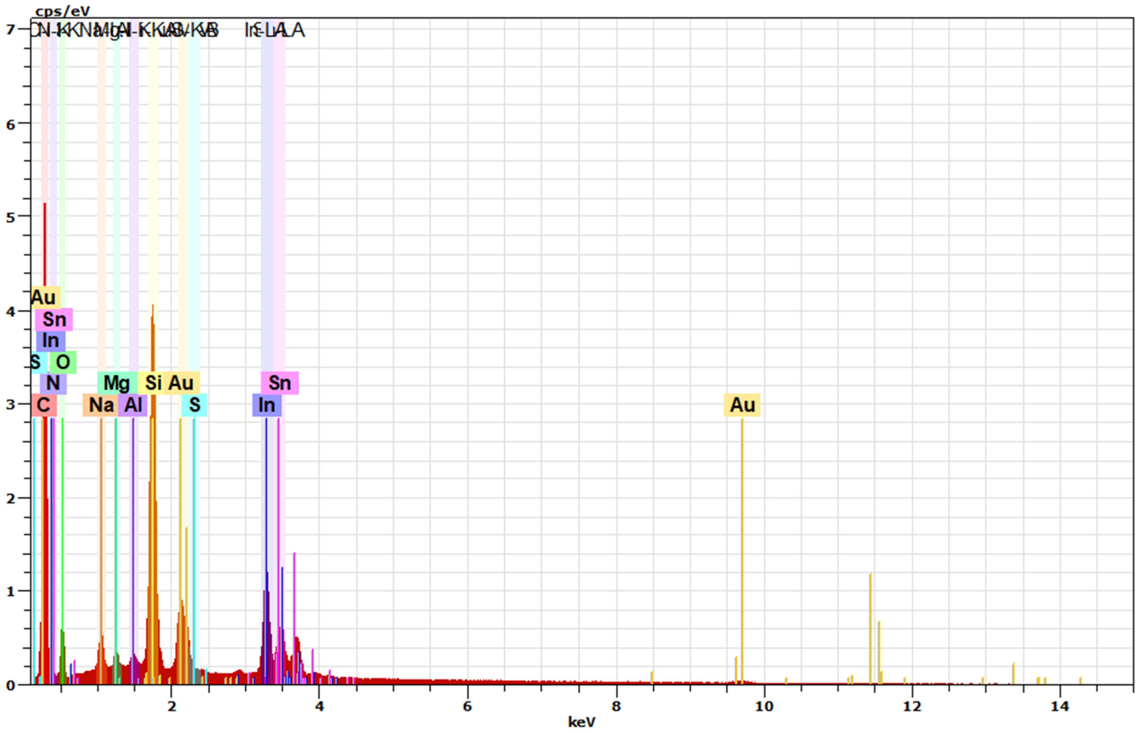

Spectrum: Map

Element	Series	unn. C [wt.%]	norm. C [wt.%]	Atom. C [at.%]	Error (1 Sigma) [wt.%]
Carbon	K-series	37.00	41.80	73.01	4.24
Nitrogen	K-series	0.00	0.00	0.00	0.00
Oxygen	K-series	6.12	6.91	9.06	0.87
Sodium	K-series	1.41	1.59	1.45	0.12
Magnesium	K-series	0.47	0.53	0.46	0.05
Aluminium	K-series	0.36	0.40	0.31	0.04
Silicon	K-series	11.82	13.36	9.98	0.52
Sulfur	K-series	0.06	0.07	0.05	0.03
Indium	L-series	17.77	20.08	3.67	0.57
Tin	L-series	4.98	5.63	0.99	0.18
Gold	M-series	8.52	9.62	1.02	0.35
	Total:	88.51	100.00	100.00	

AuNSs@GONaOH – content of gold equals to 0.11% ± 0.07

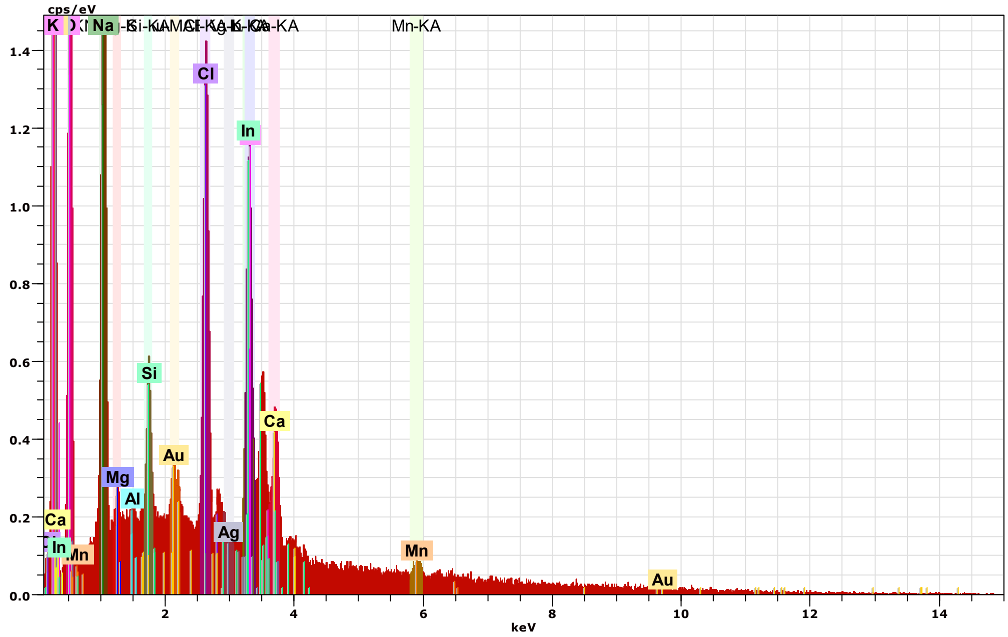

Spectrum: Map

Element	Series	unn. C [wt.%]	norm. C [wt.%]	Atom. C [at.%]	Error (1 Sigma) [wt.%]
Carbon	K-series	34.08	42.45	57.70	4.21
Oxygen	K-series	24.13	30.06	30.67	3.14
Sodium	K-series	6.82	8.49	6.03	0.45
Silicon	K-series	0.74	0.92	0.53	0.06
Chlorine	K-series	3.29	4.09	1.89	0.14
Silver	L-series	0.06	0.07	0.01	0.03
Gold	M-series	1.07	1.33	0.11	0.07
Aluminium	K-series	0.07	0.09	0.05	0.03
Magnesium	K-series	0.20	0.25	0.17	0.04
Potassium	K-series	1.65	2.06	0.86	0.08
Indium	L-series	6.43	8.02	1.14	0.23
Calcium	K-series	1.39	1.73	0.71	0.07
Manganese	K-series	0.34	0.43	0.13	0.05
	Total:	80.27	100.00	100.00	

AuNUs@GO – content of gold equal to 0.39 % +- 0.11

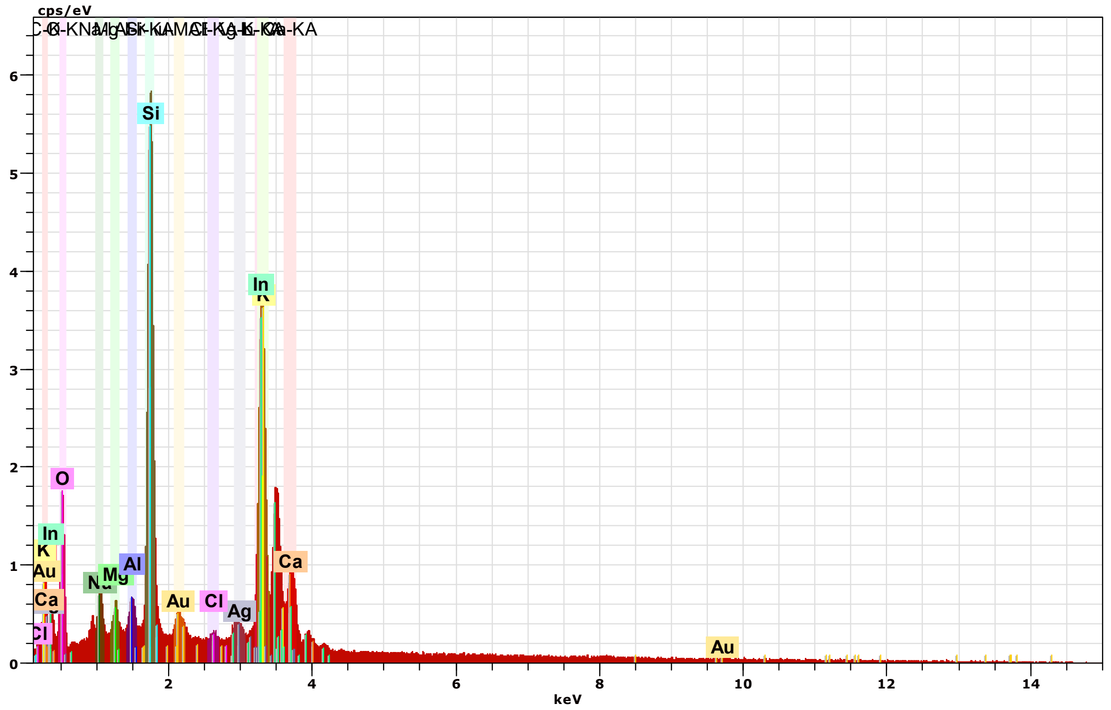


```
Spectrum: Map

Element     Series   unn. C  norm. C  Atom. C  Error (1 Sigma)
                     [wt.%]   [wt.%]   [at.%]          [wt.%]
--------------------------------------------------------------
Carbon    K-series    5.87     7.60    19.06             0.93
Oxygen    K-series   15.99    20.70    38.98             2.18
Sodium    K-series    1.79     2.31     3.03             0.14
Silver    L-series    1.69     2.18     0.61             0.09
Gold      M-series    1.97     2.55     0.39             0.11
Aluminium K-series    0.86     1.12     1.25             0.07
Silicon   K-series   12.31    15.94    17.10             0.54
Chlorine  K-series    0.19     0.25     0.21             0.04
Potassium K-series    5.75     7.45     5.74             0.21
Indium    L-series   26.61    34.45     9.04             0.84
Calcium   K-series    3.47     4.49     3.38             0.14
Magnesium K-series    0.75     0.97     1.21             0.07
--------------------------------------------------------------
            Total:   77.25   100.00   100.00
```

AuNUs@GONH$_3$ – content of gold equal to 0.30 %+- 0.15

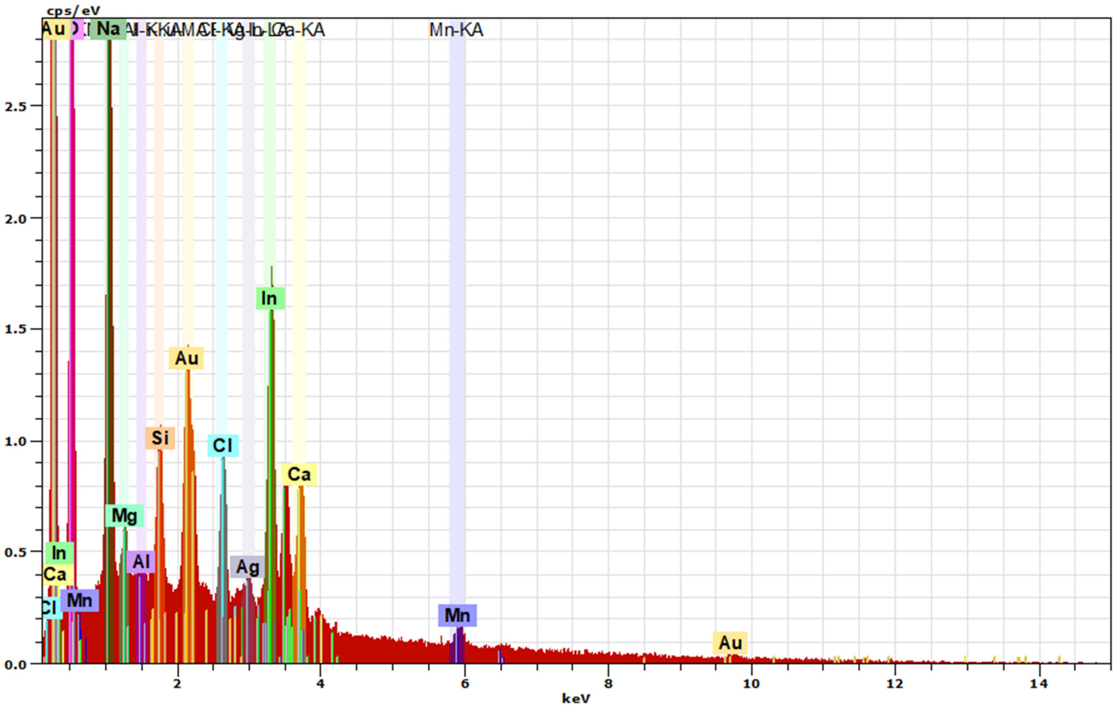

Spectrum: Map

Element	Series	unn. C [wt.%]	norm. C [wt.%]	Atom. C [at.%]	Error (1 Sigma) [wt.%]
Carbon	K-series	39.85	44.52	59.44	4.48
Oxygen	K-series	30.27	33.82	33.90	3.53
Sodium	K-series	4.06	4.53	3.16	0.28
Magnesium	K-series	0.24	0.27	0.18	0.04
Aluminium	K-series	0.02	0.02	0.01	0.03
Silicon	K-series	0.59	0.66	0.38	0.05
Chlorine	K-series	0.91	1.02	0.46	0.06
Calcium	K-series	1.52	1.70	0.68	0.07
Manganese	K-series	0.69	0.78	0.23	0.05
Silver	L-series	0.36	0.40	0.06	0.04
Indium	L-series	7.66	8.56	1.20	0.26
Gold	M-series	3.32	3.71	0.30	0.15
	Total:	89.51	100.00	100.00	

AuNUs@GONaOH – content of gold equal to 0.09 %+- 0.07

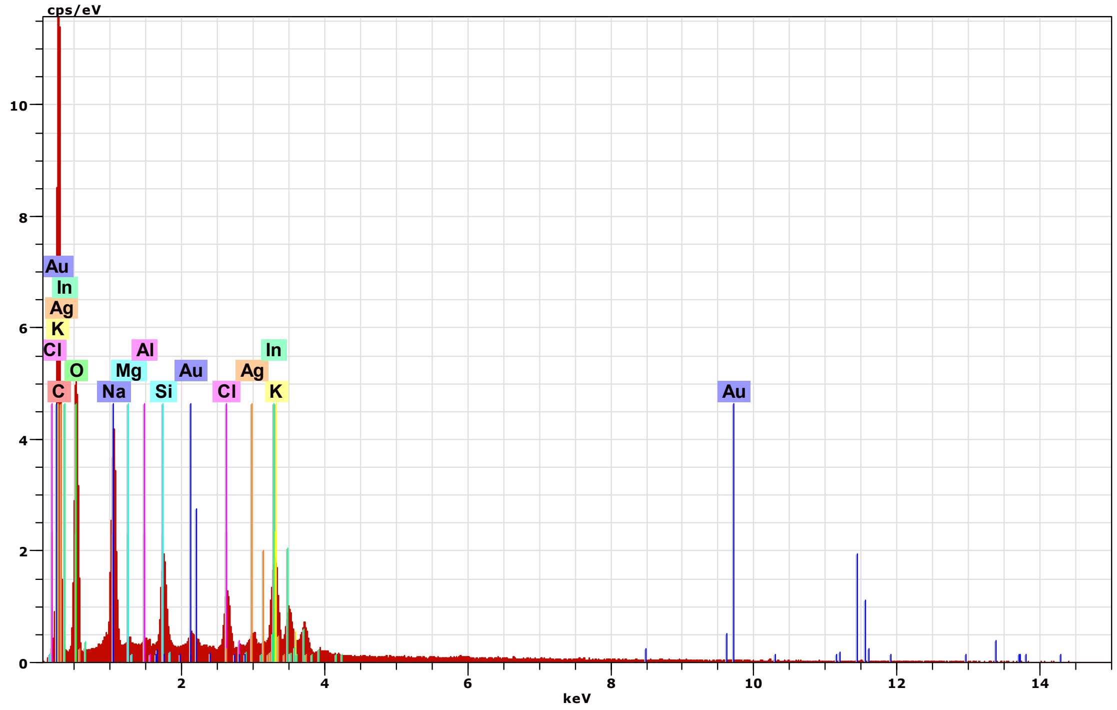

Spectrum: Acquisition

Element	Series	unn. C [wt.%]	norm. C [wt.%]	Atom. C [at.%]	Error (1 Sigma) [wt.%]
Carbon	K-series	38.59	45.30	61.16	4.58
Oxygen	K-series	24.17	28.38	28.77	3.08
Sodium	K-series	6.28	7.37	5.20	0.42
Silicon	K-series	2.03	2.39	1.38	0.11
Chlorine	K-series	1.78	2.09	0.95	0.09
Potassium	K-series	1.36	1.60	0.66	0.07
Silver	L-series	1.50	1.77	0.27	0.08
Indium	L-series	8.32	9.77	1.38	0.29
Gold	M-series	0.96	1.13	0.09	0.07
Magnesium	K-series	0.12	0.14	0.09	0.03
Aluminium	K-series	0.06	0.07	0.04	0.03
	Total:	85.17	100.00	100.00	

AuNBs@GO content of gold equal to 0.58 % ± 0.18

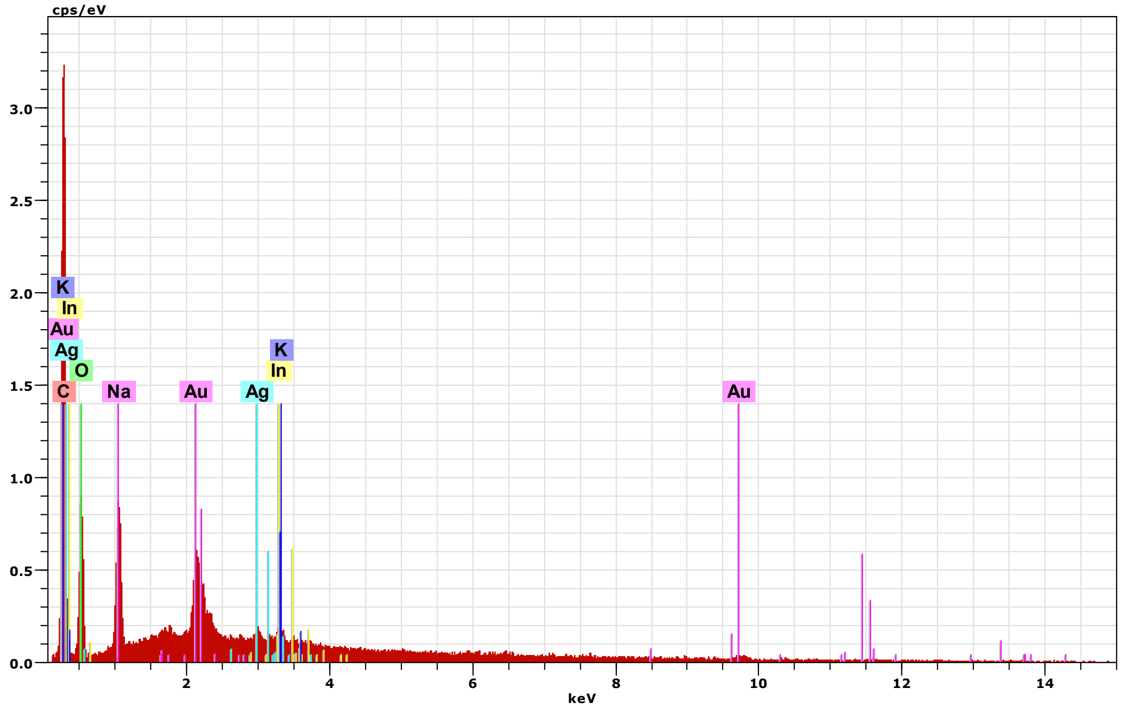

Spectrum: Acquisition

Element	Series	unn. C [wt.%]	norm. C [wt.%]	Atom. C [at.%]	Error (1 Sigma) [wt.%]
Carbon	K-series	27.15	55.58	69.35	3.57
Oxygen	K-series	12.50	25.59	23.97	1.96
Silver	L-series	0.57	1.16	0.16	0.05
Gold	M-series	3.72	7.63	0.58	0.18
Sodium	K-series	4.28	8.76	5.71	0.31
Indium	L-series	0.51	1.04	0.14	0.05
Potassium	K-series	0.12	0.25	0.09	0.03
	Total:	48.84	100.00	100.00	

AuNBs@GONH$_3$ – content of gold equals to 0.32 % ± 0.10

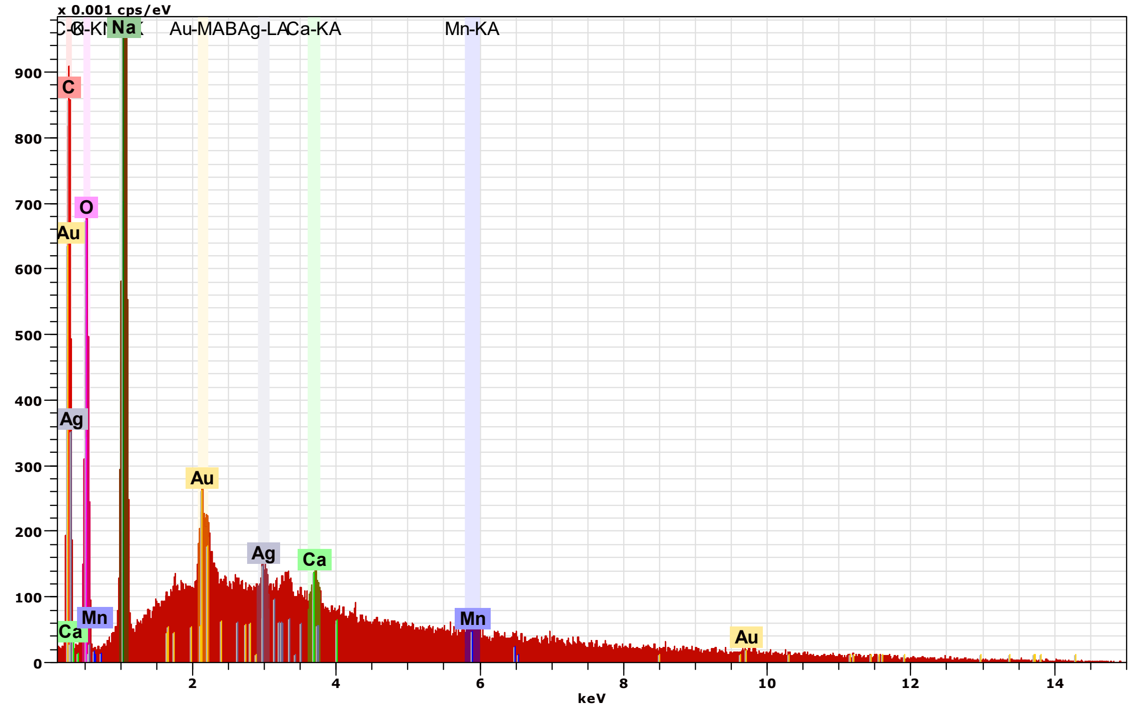

Spectrum: Map

Element	Series	unn. C [wt.%]	norm. C [wt.%]	Atom. C [at.%]	Error (1 Sigma) [wt.%]
Carbon	K-series	17.04	38.40	51.25	2.44
Oxygen	K-series	13.70	30.88	30.94	2.01
Sodium	K-series	10.72	24.17	16.85	0.70
Calcium	K-series	0.30	0.68	0.27	0.04
Manganese	K-series	0.24	0.53	0.16	0.04
Silver	L-series	0.64	1.44	0.21	0.05
Gold	M-series	1.73	3.90	0.32	0.10
	Total:	44.37	100.00	100.00	

AuNBs@GONaOH – content of gold equals to 0.29 % ± 0.10

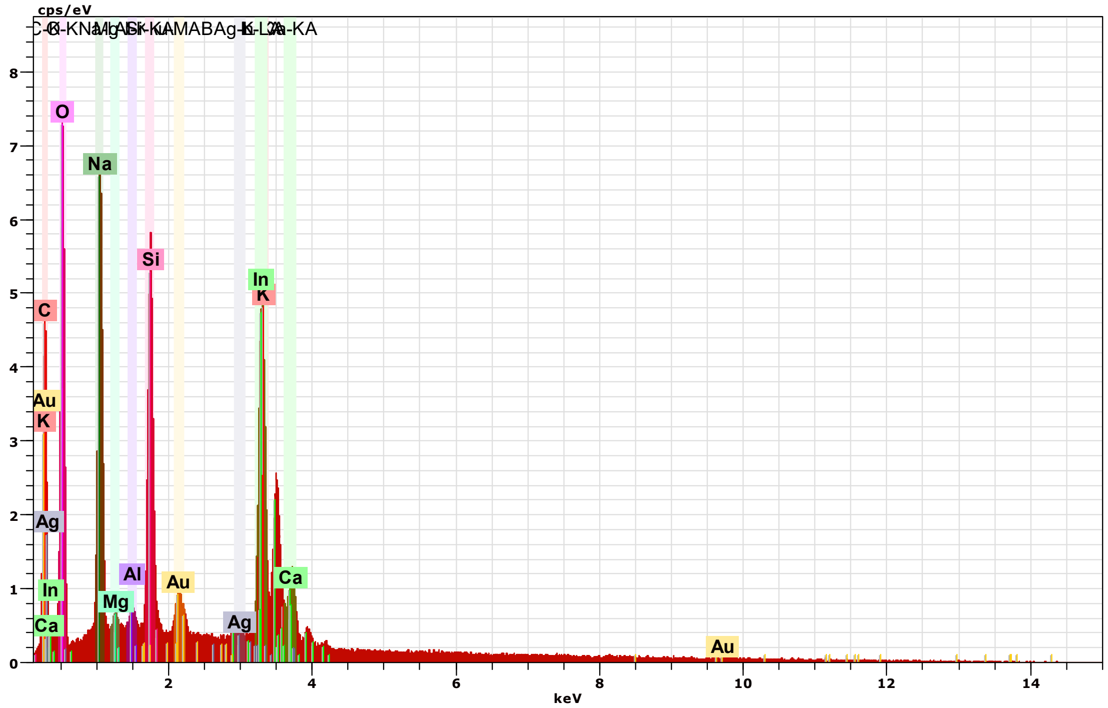

Spectrum: Map

Element	Series	unn. C [wt.%]	norm. C [wt.%]	Atom. C [at.%]	Error (1 Sigma) [wt.%]
Carbon	K-series	11.65	14.77	28.17	1.53
Oxygen	K-series	24.43	30.97	44.36	2.99
Sodium	K-series	8.91	11.30	11.26	0.58
Magnesium	K-series	0.21	0.27	0.25	0.04
Aluminium	K-series	0.26	0.33	0.28	0.04
Silicon	K-series	5.73	7.26	5.93	0.27
Potassium	K-series	4.12	5.22	3.06	0.16
Calcium	K-series	1.98	2.51	1.44	0.09
Silver	L-series	0.33	0.41	0.09	0.04
Indium	L-series	19.33	24.50	4.89	0.62
Gold	M-series	1.94	2.45	0.29	0.10
	Total:	78.89	100.00	100.00	
